# Handheld Near-Infrared Spectroscopy for Undried Forage Quality Estimation

**DOI:** 10.3390/s24165136

**Published:** 2024-08-08

**Authors:** William Yamada, Jerry Cherney, Debbie Cherney, Troy Runge, Matthew Digman

**Affiliations:** 1 Department of Biological Systems Engineering, University of Wisconsin—Madison, Madison, WI 53706, USA; trunge@wisc.edu (T.R.); digman@wisc.edu (M.D.); 2 Section of Soil and Crop Sciences, School of Integrative Plant Science, Cornell University, Ithaca, NY 14853, USA; jhc5@cornell.edu (J.C.); djc6@cornell.edu (D.C.)

**Keywords:** portable NIRS, miniaturization, feed composition, scanning methods

## Abstract

This study investigates the efficacy of handheld Near-Infrared Spectroscopy (NIRS) devices for in-field estimation of forage quality using undried samples. The objective is to assess the precision and accuracy of multiple handheld NIRS instruments—NeoSpectra, TrinamiX, and AgroCares—when evaluating key forage quality metrics such as Crude Protein (CP), Neutral Detergent Fiber (aNDF), Acid Detergent Fiber (ADF), Acid Detergent Lignin (ADL), in vitro Total Digestibility (IVTD)and Neutral Detergent Fiber Digestibility (NDFD). Samples were collected from silage bunkers across 111 farms in New York State and scanned using different methods (static, moving, and turntable). The results demonstrate that dynamic scanning patterns (moving and turntable) enhance the predictive accuracy of the models compared to static scans. Fiber constituents (ADF, aNDF) and Crude Protein (CP) show higher robustness and minimal impact from water interference, maintaining similar $R^{2}$ values as dried samples. Conversely, IVTD, NDFD, and ADL are adversely affected by water content, resulting in lower $R^{2}$ values. This study underscores the importance of understanding the water effects on undried forage, as water‘s high absorption bands at 1400 and 1900 nm introduce significant spectral interference. Further investigation into the PLSR loading factors is necessary to mitigate these effects. The findings suggest that, while handheld NIRS devices hold promise for rapid, on-site forage quality assessment, careful consideration of scanning methodology is crucial for accurate prediction models. This research contributes valuable insights for optimizing the use of portable NIRS technology in forage analysis, enhancing feed utilization efficiency, and supporting sustainable dairy farming practices.

## 1. Introduction

Understanding and managing the nutritional variability of forages is crucial for optimizing dairy farm management and enhancing animal health. The nutritive value of forages, including alfalfa-grass haylage and corn silage, exhibits considerable variability, which can influence milk production efficiency and environmental sustainability [1]. Recent advancements have leveraged handheld near-infrared (NIR) spectroscopy devices, such as the NeoSpectra (1350–2550 nm, Si-ware Systems Inc., Cairo, Egypt), TrinamiX (1450–2450 nm, TrinamiX Inc., Ludwigshafen, Germany), and Agrocares Scanners F-Series (1300–2550 nm, AgroCares, Wageningen, The Netherlands). These tools offer rapid on-site prediction of forage quality, enabling nutritional management by detecting variations in forage nutritive values, including dry matter (DM) [2,3,4,5], Crude Protein (CP) [2,3,4,5,6,7,8,9,10], and fiber contents and properties, such as actual Neutral Detergent Fiber (aNDF) [2,6,7,8,9,10], Neutral Detergent Fiber Digestibility (NDFD) [4,7,8], Acid Detergent Fiber (ADF) [2,3,4,6,7,8,9,10], Acid Detergent Lignin (ADL) [2,4,7,8], and in vitro Total Digestibility (IVTD) [4,6,7,8,10]. By facilitating daily adjustments to animal diets based on accurate, real-time forage analysis, handheld NIR devices can significantly enhance the efficiency of feed utilization, reduce environmental impact, and improve the overall profitability and sustainability of dairy farming operations [3,11,12].

The interest in compact, handheld spectrometers for analyses conducted directly on farms is gaining momentum [2,13,14]. These NIR devices have proven to have good performance on pre-treated samples of dried and ground forage [6]. These portable spectrometers are becoming more common on machinery used for forage harvesting and have also been modified for use with other agricultural implements like liquid manure spreaders [15,16]. In a study [17], it was found that a portable device (HarvestLab^TM^ 3000, Deere & Company, Moline, IL, USA) could approximate the quality of a mix of undried forage species, albeit with systematic errors that could be adjusted for accuracy. An essential element for the effectiveness of NIR technology in these applications is the development of a reliable calibration.

The utilization of handheld Near-Infrared Spectroscopy (NIRS) devices in forage assessment aims to permit direct sample analysis without the need for prior sample processing. This is enabled by calibrations developed for wet, unprocessed forage. However, deploying these devices faces several obstacles, notably the influence of the moisture absorption band and sample heterogeneity [18,19]. Additional operational challenges of handheld NIRS technology include managing the signal-to-noise ratio, ensuring a consistent power supply in a portable format, and maintaining functionality amid diverse and potentially adverse environmental conditions [3,7].

These studies underscore the practical considerations and performance of handheld NIRS instruments in the field. For instance, [7] describes the development of predictive models for various forage types, highlighting the significant influence of the scanning methodology on the accuracy of the constituent prediction. Similarly, ref. [3] provides an assessment of multiple handheld NIR devices, examining their precision and accuracy in on-farm forage evaluation, with a focus on dry matter content compared to traditional moisture meters and the robustness of available calibrations for nutritive value determination.

The ongoing development of NIRS technology for forage quality prediction indicates that miniaturized instruments have similar predictive power as benchtop instruments. As this technology is increasing in popularity, it is essential to understand if different portable instruments and scanning patterns affect the quality of the prediction. Thus, the objectives of this research are as follows:

- To assess the precision and accuracy of multiple handheld Near-Infrared Spectroscopy (NIRS) devices when used for on-farm forage evaluation, particularly focusing on the robustness of calibrations for nutritive value determination;
- To examine if different portable instruments and scanning patterns influence the quality of prediction;
- To evaluate the effects of using dried unground samples for forage quality prediction.

## 2. Materials and Method

### 2.1. Samples and Reference Analysis

Predictive NIRS models were developed using NIRS spectra and laboratory reference values for 600 silage samples of mixed haylage. Silage samples were collected between 2021 and 2023 from silage bunkers on 111 farms around New York State. After collection, the samples were vacuum-packed in oxygen-limiting polyethylene bags using a commercial vacuum packing machine for scanning at a later date.

The acquisition of NIR spectroscopic measurement data was achieved using three scanners: NeoSpectra (1350–2550 nm, Si-ware Systems Inc., Cairo, Egypt), TrinamiX (1450–2450 nm, TrinamiX Inc., Ludwigshafen, Germany), and AgroCares (1300–2550 nm, AgroCares, Wageningen, The Netherlands). The data collected with TrinamiX and AgroCares reported spectra from 1454 to 2446 nm at a fixed step of 4 nm, while the NeoSpectra scanner reported spectra from 1350 to 2550 nm at a variable step between 2.5 and 8.8 nm and a wavelength resolution of 16 nm. Each scanner used different detector types as shown in Table 1.

Before the scanning process began, all samples were thoroughly mixed in a large plastic container to ensure homogeneity. All samples were scanned in a controlled laboratory environment to avoid any interference of humidity or temperature on the scans. Two primary methods were employed to capture spectra, alongside a third specialized technique. The first method involved placing the scanner‘s lens in direct contact with the sample, where it remained stationary. The second method required the scanner to be moved across the sample surface during the scanning period, maintaining continuous contact. After each scan conducted with the second method, the samples were mixed again to ensure consistency; this method was tested with the AgroCares and NEOSpectra scanners. The third technique utilized a rotating dish accessory (Si-ware Systems Inc., Cairo, Egypt) for the NEOSpectra instrument, allowing the sample to be scanned continuously. These methods were sequentially applied to each forage sample, with five replicate scans being collected to ensure accuracy and repeatability.

Post-acquisition, the samples were desiccated using forced-air ovens until a consistent mass was achieved at 60 °C, subsequently ground with a Wiley mill (Thomas Scientific, Swedesboro, NJ, USA) through a 1 mm mesh screen, and then stored in plastic containers. The forage constituents appraised included Neutral Detergent Fiber (aNDF), in vitro Total Digestibility (IVTD), Neutral Detergent Fiber Digestibility (NDFD), Acid Detergent Lignin (ADL), Acid Detergent Fiber (ADF), ash, and Crude Protein (CP), which served as reference variables for the calibration of Near-Infrared Spectroscopy (NIRS) predictive models.

### 2.2. Wet Chemistry

For the chemical analysis, the methodologies aligned with those delineated in the literature [20]. Concisely, forage samples were apportioned into ANKOM F57 filter bags (ANKOM Technology, Macedon, New York, NY, USA) to quantify NDF, ADF, ADL, and 48-h IVTD. To alleviate gaseous pressure, the filter bags were intermittently removed from their respective containers on both the initial and subsequent days. The digestibility of the Neutral Detergent Fiber was quantified in terms of the percentage of fiber hydrolyzed, with the values expressed on an NDF basis.

The nitrogen (N) content was measured through a combustion technique using a LECO CN628 analyzer (DairyOne, Ithaca, NY, USA), with Crude Protein (CP) being inferred from nitrogen values using the conversion factor of 6.25 as per AOAC guidelines (1995). Duplicate analyses were performed for all constituents, with nitrogen content being quantified in duplicate on a select sample subset to establish the Standard Error for the CP measurement. The laboratory‘s Standard Error (SEL) pertinent to these determinations has been documented in prior studies that used the same samples but with different instruments and scanning analysis [3,7].

### 2.3. Model Calibration

To ensure methodological consistency and mitigate the risk of overfitting, uniform data preprocessing and training protocols were employed across all instrument models. Spectral data from the NEOSpectra device were interpolated to achieve a consistent interval of 4 nm. All spectral data were converted to absorbance by employing the logarithmic transformation of the reciprocal reflectance, denoted as log(1/R). Data preprocessing was standardized using a Savitzky–Golay filter with a window length of four, a polynomial order of three, and a derivation order of one. The algorithm of choice for the modeling was Partial Least Squares Regression (PLSR), using Python 3.10.12 and the packages scipy (to preprocess the data) and scikit-learn (to calibrate the PLSR model). In order to have a baseline model for each predicted variable, we opted to work with PLS-1 to understand how the scans affected the individual performance of the models. The selection of the optimal number of latent variables within the range of 1 to 20 was systematically determined using a grid search.

The dataset was divided into 90%/10% for training with five-fold cross-validation (CV) and a separate validation dataset, respectively. We randomly selected bunkers to split the dataset, ensuring that the training and test sets were independent. This approach uses 540 samples for calibration (432 for training and 108 for CV) and 60 samples for testing the final model, providing a robust evaluation of the model‘s generalizability. By having an external dataset for validation, created as described, we can effectively verify the overfitting of our model [21]. Overfitting can be identified by comparing performance metrics between the training and validation datasets. If the model performs significantly better on the training data than on the validation data or the validation performance deteriorates while the calibration improves, it is likely overfitting. In our study, we adopt a robust outlier detection method utilizing Partial Least Squares (PLS) regression tailored for Near-Infrared (NIR) spectroscopy data analysis. This approach leverages Q-residuals and Hotelling‘s T-squared statistics to identify deviations, ensuring outliers that could skew the model‘s predictive accuracy are effectively recognized, using a 95% confidence level [22]. This technique provides a systematic way to refine datasets for better analytical outcomes.

### 2.4. Evaluation

The calibration models were evaluated using the standards set forth by Malley et al. [23] and Williams et al. [24] as shown in Table 2. This section details the predictive performance of the models using a suite of metrics, including root mean square error (RMSE), Bias, Standard Error (SE), Cross-Validation Standard Error (SECV), Coefficient of Determination ($R^{2}$), Cross-Validated $R^{2}$ ($R_{CV}^{2}$), Ratio of Performance to Deviation (RPD), Cross-Validated RPD ($RPD_{CV}$), and the number of latent variables (LVs) employed on the PLS calibration.

We will compare our results with those obtained from various handheld NIRS devices reported in the literature. Specifically, we will reference studies that utilized different instruments on both dried and undried materials. For undried and unground material, we will compare our findings with those from the Aurora device as reported by Cherney et al. (2021) [3] and the NEOSpectra device as reported by Feng et al. (2023) [7]. For dried and ground material, comparisons will be made with results obtained using the MicroPHAZIR (1600–2400 nm, ThermoFisher Scientific, Waltham, MA, USA) and DLP NIRscan Nano EVM (900–1700 nm, Texas Instruments, Dallas, TX, USA) devices as described by Acosta et al. (2020) [6], the NEOSpectra device as reported by Digman et al. (2022) [4], the ASD QualitySpec (350–2500 nm, Malvern Panalytical, Cambridge, UK) and Tellspec (900–1700 nm, Tellspec Inc., Toronto, ON, Canada) devices as detailed by Rukundo et al. (2021) [2], and the Aurora (950–1650 nm, GraiNit S.r.l., Padua, Italy), NIR-S-G1 (950–1650 nm, InnoSpectra, Hsinchu, Taiwan), and SCiO (740–1070 nm, Consumer Physics, Hod Hasharon, Israel) devices as discussed by Berzaghi et al. (2021) [8]. This comprehensive comparison will provide a robust context for evaluating the performance and accuracy of our results and evaluating the impact of using undried materials. In addition, to evaluate the effects of water absorption, we will analyze the major PLS loading components in relation to the water absorption bands as described by Williams [25]. The primary water absorption bands are detailed in Table 3.

## 3. Results and Discussion

### 3.1. Spectral Data

**Figure 1:**
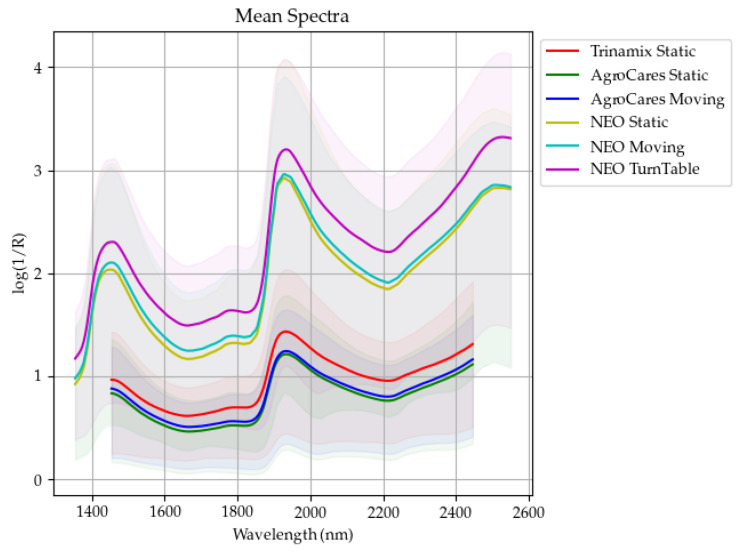
Comparative analysis of forage sample spectra: This graph illustrates the mean spectral signatures of forage samples (*n* = 600) as measured by three different scanners—TrinamiX (red line—static scan), AgroCares (green line—static scan; blue line—moving scan), and NEOSpectra (yellow—static scan; cyan—moving scan; magenta—turntable scan)—utilizing varying methods. Each line represents the average log(1/R) value across a range of wavelengths from 1400 to 2600 nm. The hue of each line represents the range between the maximum and minimum measured for each instrument.

The average spectra and range of the 600 samples scanned are shown in Figure 1, which presents the mean spectral signatures captured by each instrument. In accordance with the observations reported by Feng et al. [7], overtone bands attributable to O-H bonds are discernible at approximately 1400 and 1900 nm, which is consistent with the presence of moisture in the undried forage samples. The statistical information of the laboratory measurements of the constituents is provided in Table 4.

### 3.2. Calibration Results

The calibration outcomes are summarized in Table 5. The NEOSpectra device in turntable mode yielded superior fit models for the calibration set variables ADF, ADL, CP, and aNDF. With respect to IVTD and NDFD, this device also demonstrated superior performance in certain metrics while remaining competitive in others. An observation is that both the moving and turntable scanning modes achieved the best calibration results when utilizing a greater number of latent variables, suggesting that dynamic scanning captures more relevant data for model calibration.

**Figure 2:**
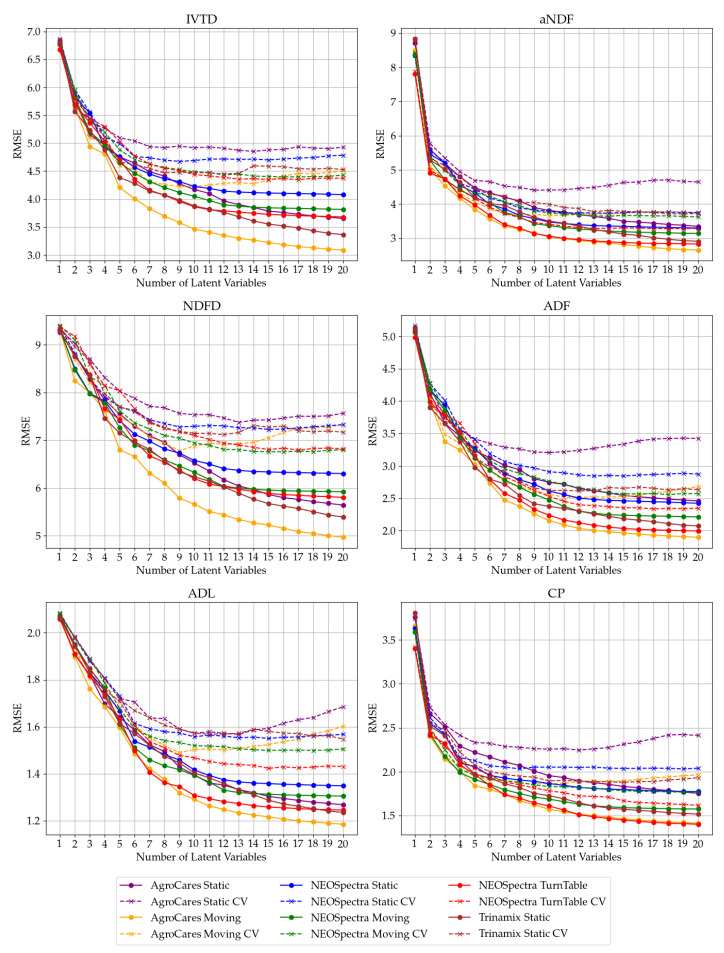
RMSE vs. latent variables for each variable. This figure shows the root mean squared error (RMSE) values for different numbers of latent variables across various instruments and target variables. The RMSE values for both calibration and cross-validation (CV) are plotted for each instrument, differentiated by color (purple—AgroCares Static, orange—Agrocares Moving, blue—NEOSpectra Static, green—NEOSpectra Moving, red—NEOSpectra Turntable, brown—Trinamix Static) and line style (continuous—calibration, dashed—CV).

Figure 2 illustrates the relationship between the number of latent variables (LVs) and the root mean square error (RMSE) for various instruments and target variables. The analysis reveals that dynamic scans, represented by moving and turntable configurations, exhibit less sensitivity to larger number of latent variables. In contrast, static scans demonstrate signs of overfitting when more than 10 latent variables are used as indicated by the deterioration in CV performance, evidenced by increasing the RMSE, despite improvements in the calibration RMSE. This observation underscores the importance of scan dynamics in mitigating overfitting and enhancing the predictive accuracy of models across different latent variable configurations. The explained variability of the LVs can be found in Table A1 in Appendix A. Overall, the improvements using more latent variables are not that significant for more than 10 LVs, achieving less than one percent improvement in the RMSE.

### 3.3. Validation Results

**Figure 3:**
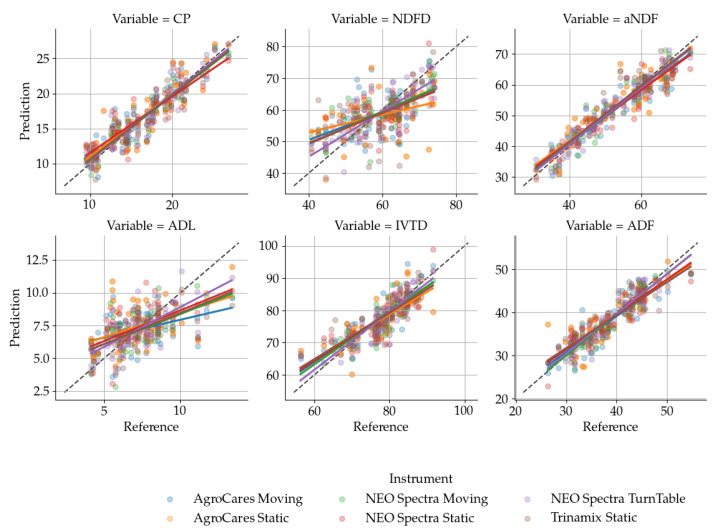
Comparative evaluation of three handheld spectrometers and methods used to predict nutritional content in feed samples. The different colors and shapes represent readings from moving, static, or turntable methods of using the AgroCares, NEO Spectra, and TrinamiX instruments. Each dot represents the pair of reference data and the prediction using the calibrated PLSR model from the validation set (*n* = 60). The regression lines for each method showcase the accuracy and precision in predicting the content of Crude Protein (CP), Neutral Detergent Fiber (aNDF), Acid Detergent Fiber (ADF), Acid Detergent Lignin (ADL), Neutral Detergent Fiber Digestibility (NDFD), and in vitro Total Digestibility (IVTD). The dashed black line represents a 1:1 agreement between the reference and predicted values.

The validation results are reported in Table 6 and summarized in Table 7, the performance of the NEOSpectra and Trinamix instruments across various modes and variables is quantified through metrics such as RMSE, SE, $R^{2}$, and RPD. For the NEOSpectra instrument, when operating in ‘Moving‘ mode, CP predictions were moderately successful ($R^{2}=0.892$) with a corresponding RPD of 3.042, leading to a ‘Good‘ classification. However, the same instrument‘s performance predicting IVTD in the same mode was not useful, with a lower $R^{2}$ of 0.743 and an RPD of 1.974, reflecting a ‘Very poor’ classification. When utilizing the ‘Turntable’ mode, predictions of ADF and ADL yielded ‘Fair’ and ‘Very poor’ classifications, respectively, indicating varied efficacy based on the forage constituent analyzed. In contrast, the Trinamix instrument in ‘Static’ mode demonstrated ‘Successful’ prediction for aNDF with an $R^{2}$ of 0.916 and an RPD of 3.452, garnering a ‘Very good’ classification. These results reflect the nuanced capabilities of each instrument and mode combination, emphasizing the importance of selecting the appropriate setup for specific analytical needs in forage assessment. Figure 3 makes it clear that the calibrated models did not perform well in predicting NDFD, ADL, and IVTD on the validation set for undried haylage samples.

When calibrated exclusively with static scans, TrinamiX had a better RMSE, SE, and RPD than the NEOSpectra for predicting CP and ADL. These findings concur with the insights of Feng et al. [7], highlighting the enhanced spectral quality afforded by moving scans due to their capacity to encapsulate a more generalized and homogeneous representation of the samples. A closer examination of the calibration data delineated in Table 5 reveals a performance hierarchy within the same instrument, with the order of efficacy being turntable > moving > static. This sequence also correlates with the increasing number of latent variables that can be utilized in the NEOSpectra scanning process, thereby suggesting that sliding scans not only improve the spectral quality but also allow for a better model calibration.

Utilizing the same scanning pattern—whether static, moving, or turntable—tends to yield comparable calibration performance across different devices as evidenced by the minimal variation in the $R^{2}$ and the RPD values, usually within the same class range of success, according to Table 2. This is consistent across most variables, with the notable exception of Neutral Detergent Fiber (aNDF), where the Trinamix instrument in static mode achieved an RPD of 3.453, surpassing those of AgroCares at 2.506 and NEOSpectra at 2.864. The findings thus suggest that the methodology of spectral data acquisition is more important than the choice of the handheld instrument.

**Figure 4:**
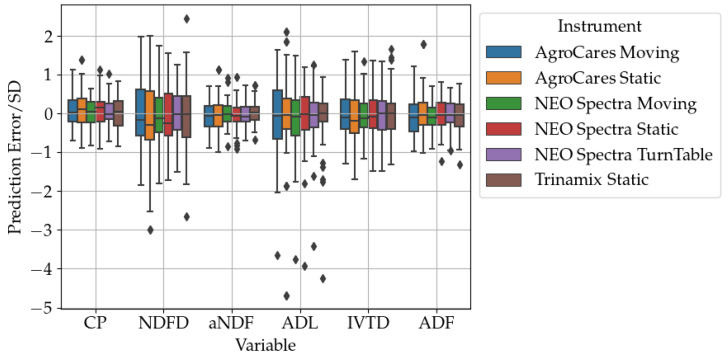
The chart presents the normalized distribution of prediction errors on the validation set for six forage quality variables—CP, NDFD, aNDF, ADL, IVTD, and ADF—obtained using different spectral scanning instruments and methods. Each boxplot shows the median, quartiles, and outliers for the prediction error standard deviation (SD) of each method.

Figure 4 presents normalized boxplots of prediction errors, facilitating a more nuanced comparison of calibration performance. Notably, CP, ADF, and aNDF demonstrate the most favorable results, characterized by minimal bias and RMSE, with most prediction errors falling within one standard deviation. Conversely, ADL predictions are less accurate, exhibiting a multitude of outliers as reflected in Table 7, indicating a disparity between the predicted and observed values.

The standard error of laboratory (SEL) values reported by Cherney et al. (2021) [3] for the wet chemistry of the same samples indicated errors that are an order of magnitude lower than the root mean square error (RMSE) values from our results presented in Table 7 ($SEL_{aNDF}\leq 0.66$, $SEL_{ADF}\leq 0.70$, $SEL_{ADL}\leq 0.30$, $SEL_{IVTD}\leq 0.76$, $SEL_{NDFD}\leq 2.36$, and $SEL_{CP}\leq 0.44$).

**Figure 5:**
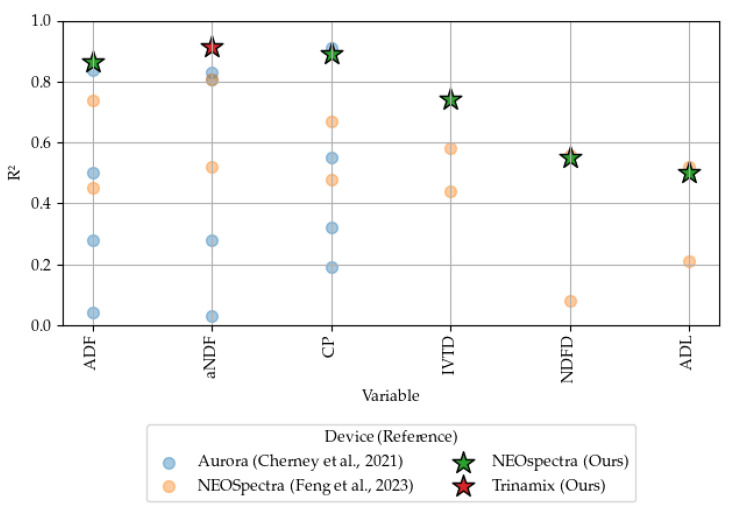
Undried data $R^{2}$ comparison with Aurora [3], calibrated for haylage, corn silage, and Total Mixed Ration, and NEOSpectra [7] calibrated for grass, alfalfa, and mixed silage forages. Both references were sampled using moving scans. The dots represents the metrics obtained by the references, and the stars represents the metrics obtained by our best model.

Compared to the existing literature, our calibration results are compatible with the previously reported findings. For undried samples, [3] evaluated the Aurora instrument for haylage, corn silage, and Total Mixed Ration, while [7] assessed the NEOSpectra for corn silage, alfalfa, grass, and mixed alfalfa and grass silage. Both studies utilized moving scans. As shown in Figure 5, our results with haylage samples exhibit similar characteristics to the best metrics from the literature, further supporting the notion that dynamic scans often outperform other methods. Additionally, our findings indicate that ADF, aNDF, and CP achieve moderately successful-to-excellent calibrations, whereas IVTD, NDFD, and ADL do not yield useful models according to the [23] $R^{2}$ criteria.

**Figure 6:**
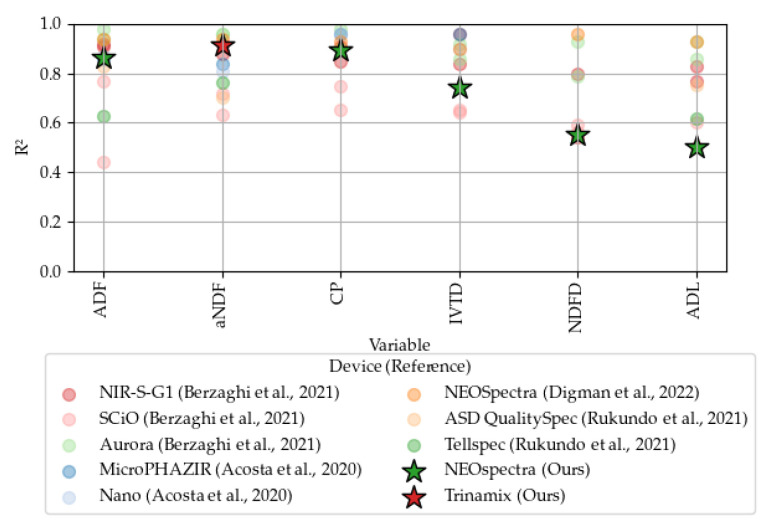
Comparison of $R^{2}$ values from models calibrated on dried samples (literature) versus our model calibrated on undried samples. Tellspec and ASD QualitySpec [2] were calibrated for grass. NEOSpectra [4] was calibrated for grass, alfalfa, and mixed silage forages. Nano and MicroPHAZIR [6] were calibrated for grass forages. NIR-S-G1, SCiO, and Aurora [8] were calibrated for alfalfa and grass forages. The dots represent the metrics obtained from the references, and the stars represent the metrics obtained by our best model.

As one of the goals of portable NIRS is to be used for in-field forage quality estimation, it is important to understand how the prediction model performance is affected when using undried samples. Since water has high absorption bands at 1400 and 1900 nm, it creates interference in the raw spectrum of the material in the NIR region [25]. Figure 6 illustrates how our model performance compares to that of models calibrated on dried samples. It is evident that the water content of the samples affects the performance of the models to varying degrees for all forage quality metrics studied. ADF, aNDF, and CP are less impacted by water, exhibiting similar $R^{2}$ values to those of dried materials. In contrast, IVTD, NDFD, and ADL are severely affected by the water content, resulting in lower $R^{2}$ values. Further investigation into how water influences the PLSR loading factors is necessary to better understand whether these effects can be mitigated.

**Figure 7:**
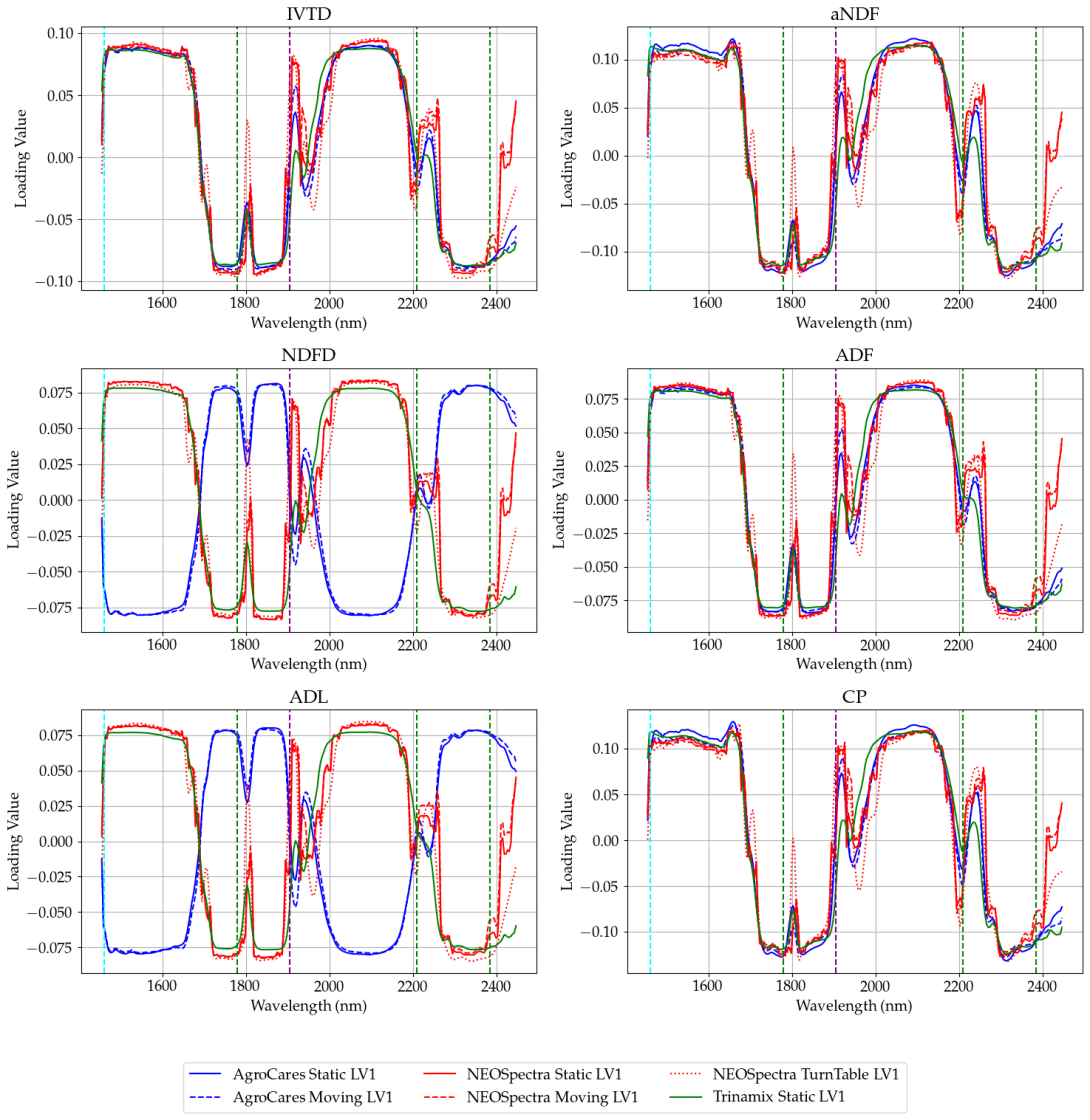
Loading values of the first latent variable of the spectrum (first derivative). Instruments are divided by color (AgroCares—blue, NEOSpectra—red, and Trinamix—green). The scan modes are divided by the line style (continuous—static, dashed—moving, and dotted—turntable). The vertical lines are the water absorption bands. Very small absorption bands (1778, 2208, and 2384 nm) are in green. The large absorption band (1460 nm) is illustrated in cyan. The very large absorption band (1904 nm) is shown in purple.

**Figure 8:**
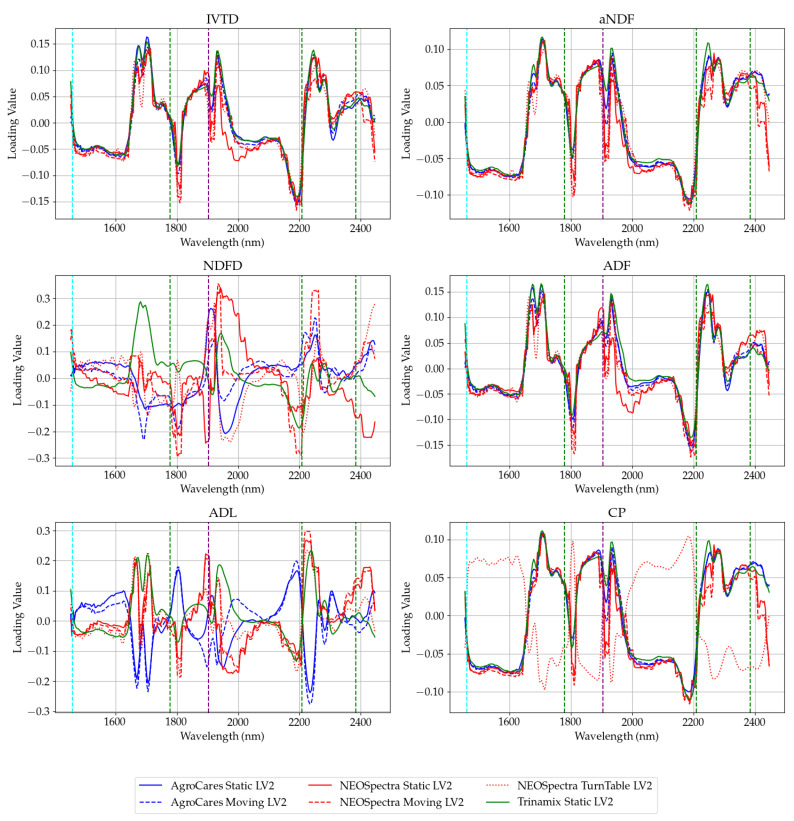
Loading values of the second latent variable of the spectrum (first derivative). Instruments are divided by color (AgroCares—blue, NEOSpectra—red, and Trinamix—green). The scan modes are divided by the line style (continuous—static, dashed—moving, and dotted—turntable). The vertical lines are the water absorption bands. Very small absorption bands (1778, 2208, and 2384 nm) are in green. The large absorption band (1460 nm) is illustrated in cyan. The very large absorption band (1904 nm) is in purple.

Figure 7 and Figure 8 display the first two latent variables that contain most of the explained variance of the PLS models (Appendix A). It is evident that the main absorption bands of water play a role in the loading factors of the latent variables, particularly at the 1904 nm band, where water has a significant absorption peak. These effects arise from the interactions of water with the O-H groups present in carbohydrates, fats, and proteins, which can form hydrogen bonds with most types of fiber. These results are consistent with the findings obtained from studies on small grains [25].

Collectively, these analyses underscore the significance of the scanning pattern over the specific technology or instrument used. The consistency in data acquisition methodology emerges as a critical factor in the calibration performance, influencing the robustness of predictive models more substantially than the hardware utilized. Furthermore, the lower performance of certain variables is likely due to the water‘s electromagnetic absorption and interaction with the undried sample material.

## 4. Conclusions

This study systematically explores the influence of the scanning methodology and instrument design on the efficacy of spectroscopic models in forage analysis. Our findings are derived from a set of 600 ensiled forage samples collected across New York state. As detailed in Table 5 and Table 6, and summarized in Table 7, we highlight the importance of the role the spectral acquisition technique plays over the specific technical features of handheld NIRS devices.

The consistency observed across the instruments when identical scanning patterns were employed underscores the methodological influence over technology. Specifically, the NEOSpectra instrument, when employed in a dynamic mode, demonstrates a significant advantage in the predictive accuracy for all variables. This suggests that the precision and reliability of predictions are more heavily contingent upon applying robust and consistent scanning protocols.

Based on the analysis of Figure 2 and Table A1, we recommend limiting the number of latent variables to 7–10 to avoid overfitting and ensure future model performance. Although our results indicate that 11–20 LVs can have a small improvement in the unseen validation set, the explained variance of these variables does not significantly improve the results enough to justify using them. Therefore, a careful balance must be struck between model complexity and predictive stability.

Comparative analyses, particularly for undried forage, have aligned with the findings from the literature, confirming the validity of our models within the expected performance parameters. Moreover, the results have revealed that scanning modes incorporating movement tend to enhance the homogeneity of the sample representation, which is critical in achieving high-quality spectral data. When comparing dried and grounded material calibrations, we can see that fiber constituents and protein are less impacted by water absorption. However, there remains a knowledge gap in understanding the water interactions of the undried and unground forage constituents, specifically how water interaction affects their NIR spectral characteristics. The loadings obtained through Partial Least Squares Regression of the NIR spectra highlight the critical role of variance at the wavelengths associated with O-H absorptions in constructing models for these materials. The behavior of water within complex agricultural substances is expected to differ from that of liquid water.

Figure 3 and Figure 4 provide visual confirmation of the comparative and error distribution analyses, respectively, illustrating the nuanced performance across different forage constituents and underscoring the models that exhibit both high accuracy and those with room for improvement.

In summary, this research affirms the importance of the scanning pattern in developing robust near-infrared spectroscopic models. It contributes valuable insights that may guide practitioners in selecting the most suitable instruments and modes for forage quality assessment. As the field advances, future studies should further refine these methodologies, optimizing the balance between technological innovation and practical application for enhanced forage analysis. The evaluation of embedded NIR sensors in agricultural machinery to predict forage quality and properties is one of the paths forward in undried forage research. 

## Figures and Tables

**Table 1:** Instruments and their characteristics.

Property	AgroCares F-Series	TrinamiX	NEO Spectra
Detector Type	MEMS	Linear Variable Filter	MEMS-FT-NIR
Spectral Range (nm)	1450–2450	1450–2450	1350–2500
Sample Scanning	Contact	Contact	Contact

**Table 2:** Calibration classification and level of success according to [23,24].

Level of Success [23]	R^2^ [23]	RPD Value [24]	Classification [24]	Application [24]
Not useful	<0.80	<2.0	Very poor	Not recommended
Moderately Successful	0.80 to 0.90	2.0 to 2.5	Poor	Rough screening
		2.5 to 3.0	Fair	Screening
Successful	0.90 to 0.95	3.0 to 3.5	Good	Quality control
		3.5 to 4.0	Very good	Process control
Excelent	>0.95	>4.0	Excellent	Any application

RPD—Ratio of Perfomance to Deviation.

**Table 3:** Positions of main absorption bands in water.

Wavelength (nm)	1460	1778	1904	2208	2384
Relative Intensity	Large	Very Small	Very Large	Very Small	Very Small

**Table 4:** Laboratory reference values statistics.

	IVTD	aNDF	NDFD	ADF	ADL	CP
Unit	%DM					
Count	600					
Mean	79.22	50.13	58.84	37.18	7.35	17.62
SD	7.31	10.42	9.39	5.80	2.18	4.43
Min	38.13	28.81	11.40	24.22	3.12	6.12
Median	80.73	48.75	58.50	36.50	7.06	18.04
Max	92.92	81.60	80.87	59.06	20.60	27.72

IVTD—in vitro Total Digestibility, aNDF—actual Neutral Detergent Fiber, NDFD—Neutral Detergent Fiber Digestibility, ADF—Acid Detergent Fiber, ADL—Acid Detergent Lignin, CP—Crude Protein, DM—Dry Matter, SD—Standard Deviation.

**Table 5:** Statistical performance metrics for calibration models using 540 samples undried and unground alfalfa samples across different instruments and modes. The table lists the RMSE, Bias, SE, SE_*CV*_, $R^{2}$, $R_{CV}^{2}$, RPD, RPD_*CV*_, and the number of latent variables (LVs) for the variables ADF, ADL, CP, IVTD, and NDFD.

Instrument	Mode	Variable	RMSE	Bias	SE	SE_*CV*_	R^2^	$R_{CV}^{2}$	RPD	RPD_*CV*_	LVs
AgroCares	Static	ADF	2.754	0.000	2.756	3.379	0.771	0.655	2.090	1.703	10
	Moving	ADF	1.959	0.000	1.961	2.608	0.884	0.795	2.937	2.208	15
NEOSpectra	Static	ADF	2.463	0.000	2.465	2.885	0.817	0.749	2.336	1.996	12
	Moving	ADF	2.122	0.000	2.124	2.544	0.864	0.805	2.711	2.264	20
	Turntable	ADF	**1.861**	0.000	**1.862**	**2.198**	**0.895**	**0.854**	**3.093**	**2.620**	19
Trinamix	Static	ADF	2.261	0.000	2.263	2.662	0.846	0.786	2.545	2.163	13
AgroCares	Static	ADL	1.365	0.000	1.367	1.674	0.591	0.386	1.564	1.277	11
	Moving	ADL	1.293	0.000	1.294	1.559	0.633	0.468	1.651	1.371	10
NEOSpectra	Static	ADL	1.369	0.000	1.371	1.592	0.589	0.445	1.559	1.342	11
	Moving	ADL	1.242	0.000	1.244	1.460	0.661	0.533	1.718	1.464	18
	Turntable	ADL	**1.175**	0.000	**1.176**	**1.450**	**0.697**	**0.539**	**1.817**	**1.473**	20
Trinamix	Static	ADL	1.405	0.000	1.406	1.608	0.567	0.434	1.520	1.329	10
AgroCares	Static	CP	2.010	0.000	2.012	2.306	0.792	0.727	2.195	1.915	9
	Moving	CP	1.670	0.000	1.672	1.872	0.857	0.820	2.641	2.358	8
NEOSpectra	Static	CP	1.843	0.000	1.845	2.140	0.825	0.765	2.393	2.063	11
	Moving	CP	1.513	0.000	1.514	1.799	0.882	0.834	2.916	2.454	20
	Turntable	CP	**1.328**	0.000	**1.329**	**1.601**	**0.909**	**0.869**	**3.322**	**2.758**	20
Trinamix	Static	CP	1.643	0.000	1.645	1.900	0.861	0.815	2.684	2.324	12
AgroCares	Static	IVTD	4.279	0.000	4.283	5.016	0.660	0.533	1.714	1.463	9
	Moving	IVTD	3.465	0.000	3.469	**4.177**	0.777	**0.677**	2.117	**1.758**	10
NEOSpectra	Static	IVTD	4.114	0.000	4.118	4.632	0.686	0.602	1.783	1.585	10
	Moving	IVTD	3.665	0.000	3.668	4.455	0.750	0.632	2.002	1.648	19
	Turntable	IVTD	**3.409**	0.000	**3.412**	4.195	**0.784**	0.674	**2.152**	1.750	20
Trinamix	Static	IVTD	3.757	0.000	3.760	4.372	0.738	0.645	1.953	1.679	12
AgroCares	Static	NDFD	6.534	0.000	6.540	7.744	0.524	0.333	1.450	1.224	10
	Moving	NDFD	5.653	0.000	5.658	6.862	0.644	0.476	1.676	1.382	10
NEOSpectra	Static	NDFD	6.317	0.000	6.323	7.173	0.555	0.428	1.500	1.322	11
	Moving	NDFD	5.730	0.000	5.735	6.980	0.634	0.458	1.653	1.358	19
	Turntable	NDFD	5.485	0.000	5.490	**6.508**	0.665	**0.529**	1.727	**1.457**	19
Trinamix	Static	NDFD	**5.390**	0.000	**5.395**	7.081	**0.676**	0.442	**1.757**	1.339	20
AgroCares	Static	aNDF	3.811	0.000	3.814	4.370	0.863	0.820	2.700	2.356	10
	Moving	aNDF	3.256	0.000	3.259	3.829	0.900	0.862	3.159	2.689	8
NEOSpectra	Static	aNDF	3.243	0.000	3.246	3.752	0.901	0.867	3.172	2.744	15
	Moving	aNDF	3.007	0.000	3.010	3.573	0.915	0.880	3.421	2.881	20
	Turntable	aNDF	**2.605**	0.000	**2.608**	**3.031**	**0.936**	**0.913**	**3.949**	**3.397**	20
Trinamix	Static	aNDF	2.905	0.000	2.908	3.828	0.920	0.862	3.541	2.689	20

SE—Standard Error, RPD—Ratio of Performance to Deviation, CV—Cross-Validated, LVs—Latent Variables, ADF—Acid Detergent Fiber, ADL—Acid Detergent Lignin, CP—Crude Protein, IVTD—in vitro Total Digestibility, NDFD—Neutral Detergent Fiber Digestibility, aNDF—actual Neutral Detergent Fiber.

**Table 6:** Validation performance metrics for different instruments operating in static, moving, and turntable modes. The metrics include RMSE, Bias, SE, $R^{2}$, Slope, Intercept, and RPD for the validation of variables ADF, ADL, CP, IVTD, NDFD, and aNDF using a set of 60 samples. This table facilitates the comparison of model precision and prediction accuracy across diverse instruments and scanning configurations for the validation dataset.

Instrument	Mode	Variable	RMSE	Bias	SE	R^2^	Slope	Intercept	RPD
AgroCares	Static	ADF	2.949	−0.150	2.970	0.761	0.973	1.191	2.047
	Moving	ADF	3.015	−0.654	2.968	0.751	0.928	3.386	2.003
NEOSpectra	Static	ADF	2.490	−0.203	2.502	0.830	1.026	−0.805	2.425
	Moving	ADF	2.283	−0.446	2.258	0.857	0.923	3.374	2.645
	Turntable	ADF	**2.207**	−0.193	**2.217**	**0.866**	0.946	2.269	**2.736**
Trinamix	Static	ADF	2.536	−0.332	2.536	0.824	1.090	−3.124	2.381
AgroCares	Static	ADL	2.400	−0.040	2.420	0.109	0.644	2.705	1.059
	Moving	ADL	2.247	−0.251	2.252	0.219	0.835	1.450	1.132
NEOSpectra	Static	ADL	2.050	−0.079	2.066	0.350	0.963	0.356	1.240
	Moving	ADL	2.013	−0.321	2.004	0.373	1.018	0.194	1.263
	Turntable	ADL	**1.794**	−0.178	**1.800**	**0.502**	1.040	−0.115	**1.417**
Trinamix	Static	ADL	1.961	−0.307	1.953	0.405	1.257	−1.550	1.297
AgroCares	Static	CP	2.003	0.356	1.988	0.783	0.893	1.408	2.144
	Moving	CP	1.729	0.329	1.712	0.838	0.937	0.700	2.484
NEOSpectra	Static	CP	1.977	0.333	1.965	0.788	0.997	−0.288	2.172
	Moving	CP	**1.412**	0.074	**1.422**	**0.892**	0.986	0.150	**3.042**
	Turntable	CP	1.517	0.140	1.524	0.875	0.935	0.911	2.831
Trinamix	Static	CP	1.712	0.101	1.723	0.841	0.925	1.112	2.509
AgroCares	Static	IVTD	4.558	−0.709	4.540	0.577	0.862	11.412	1.537
	Moving	IVTD	4.141	0.010	4.176	0.651	0.857	11.125	1.692
NEOSpectra	Static	IVTD	4.040	−0.315	4.061	0.668	0.911	7.270	1.735
	Moving	IVTD	**3.549**	−0.262	3.570	**0.743**	0.927	5.942	**1.974**
	Turntable	IVTD	3.550	−0.277	**3.569**	0.743	0.854	11.650	1.974
Trinamix	Static	IVTD	4.142	−0.180	4.173	0.651	0.912	7.068	1.692
AgroCares	Static	NDFD	8.355	−1.046	8.360	−0.019	0.497	30.314	0.991
	Moving	NDFD	7.406	−0.232	7.465	0.200	0.645	21.191	1.118
NEOSpectra	Static	NDFD	6.515	−0.590	6.543	0.381	0.838	10.082	1.271
	Moving	NDFD	5.943	−0.164	5.991	0.485	0.952	3.016	1.393
	Turntable	NDFD	**5.544**	−0.348	**5.579**	**0.552**	0.816	11.202	**1.493**
Trinamix	Static	NDFD	7.295	−0.515	7.338	0.223	0.649	21.140	1.135
AgroCares	Static	aNDF	4.380	−0.501	4.388	0.841	1.018	−0.462	2.506
	Moving	aNDF	3.808	−0.865	3.739	0.880	1.020	−0.186	2.883
NEOSpectra	Static	aNDF	3.832	−0.713	3.797	0.878	1.050	−1.935	2.864
	Moving	aNDF	3.494	−0.174	3.519	0.899	0.991	0.666	3.141
	Turntable	aNDF	3.304	−0.404	3.307	0.909	0.991	0.872	3.322
Trinamix	Static	aNDF	**3.180**	0.189	**3.201**	**0.916**	1.010	−0.705	**3.452**

SE–Standard Error, RPD–Ratio of Performance to Deviation, ADF–Acid Detergent Fiber, ADL–Acid Detergent Lignin, CP–Crude Protein, IVTD–in vitro Total Digestibility, NDFD–Neutral Detergent Fiber Digestibility, aNDF–actual Neutral Detergent Fiber.

**Table 7:** Performance of the best model for each predicted variable on the validation set.

		Variable	RMSE	SE	R^2^	RPD	Success	Classification
Instrument	Mode						($R^{2}$ [23])	(RPD [24])
NEOSpectra	Moving	CP	1.412	1.422	0.892	3.042	Moderately Successful	Good
	Moving	IVTD	3.549	3.570	0.743	1.974	Not Useful	Very poor
	Turntable	ADF	2.207	2.217	0.866	2.736	Moderately Successful	Fair
	Turntable	ADL	1.794	1.800	0.502	1.417	Not Useful	Very poor
	Turntable	NDFD	5.544	5.579	0.552	1.493	Not Useful	Very poor
Trinamix	Static	aNDF	3.180	3.201	0.916	3.452	Successful	Very good

RMSE—Root Mean Squared Error, SE—Standard Error, RPD—Ratio of Performance to Deviation, ADF—Acid Detergent Fiber, ADL—Acid Detergent Lignin, CP—Crude Protein, IVTD—in vitro Total Digestibility, NDFD—Neutral Detergent Fiber Digestibility, aNDF—actual Neutral Detergent Fiber.

## Data Availability

The data presented in this study are available on request from the corresponding author.

## Appendix A. Explained Variance

**Table A1:** Explained variance (%) by each latent variable (LV) for different instruments and target variables. The values represent the individual contribution of each LV to the total explained variance.

Instrument	Variable	LV1	LV2	LV3	LV4	LV5	LV6	LV7	LV8	LV9	LV10
AgroCares Static	ADF	69.64	9.99	1.89	5.29	2.66	2.74	1.73	2.22	0.50	0.80
	ADL	75.36	3.10	3.93	1.58	4.79	2.72	1.73	3.06	0.49	0.36
	CP	34.57	40.04	3.19	4.97	5.35	2.54	2.17	1.89	0.99	0.91
	IVTD	66.68	12.45	1.59	4.12	3.94	3.21	1.77	1.49	1.28	0.83
	NDFD	75.40	4.20	3.90	1.83	2.45	2.76	2.93	2.62	0.80	0.68
	aNDF	38.45	35.27	3.75	5.49	4.13	3.71	2.43	2.03	0.56	0.75
AgroCares Moving	ADF	72.73	11.85	1.76	5.23	2.02	1.75	0.52	1.52	0.26	0.26
	ADL	79.69	3.66	4.60	1.87	2.07	3.65	0.94	1.01	0.17	0.29
	CP	37.47	42.25	2.13	7.73	1.68	3.31	1.06	1.09	0.51	0.21
	IVTD	67.76	15.55	1.78	6.10	1.02	1.57	1.75	1.66	0.29	0.25
	NDFD	78.51	2.07	4.56	2.58	1.23	6.11	1.23	1.18	0.18	0.33
	aNDF	42.04	38.25	2.75	5.86	3.70	1.75	0.70	1.75	0.49	0.21
NEOSpectra Static	ADF	67.24	13.98	7.18	3.96	2.41	0.70	1.23	0.42	0.53	0.32
	ADL	74.43	5.09	6.78	5.11	3.41	0.64	1.10	0.63	0.44	0.31
	CP	36.26	42.49	6.67	5.75	1.79	1.20	0.63	1.28	1.00	0.45
	IVTD	62.96	17.68	5.75	5.34	1.61	1.39	1.68	0.50	0.59	0.34
	NDFD	73.31	2.32	9.77	6.12	1.84	1.48	1.91	0.47	0.43	0.35
	aNDF	36.99	38.83	10.39	4.16	3.23	0.52	1.70	0.45	0.90	0.40
NEOSpectra Moving	ADF	67.69	12.72	4.56	7.72	1.77	1.13	1.12	0.46	0.55	0.28
	ADL	73.90	4.19	8.23	5.41	2.66	0.82	0.45	0.84	1.19	0.28
	CP	35.14	39.27	3.32	13.64	2.10	0.80	1.78	0.98	0.46	0.40
	IVTD	60.10	15.80	2.24	13.97	1.72	1.27	1.47	0.54	0.60	0.28
	NDFD	72.91	1.90	7.88	9.07	2.23	0.87	1.05	1.08	0.73	0.25
	aNDF	38.82	34.14	3.75	15.01	2.22	1.30	0.78	0.85	0.33	0.59
NEOSpectra TurnTable	ADF	65.80	16.21	4.45	6.11	2.14	1.01	0.48	0.87	0.46	0.26
	ADL	72.69	6.89	6.00	3.80	5.30	1.02	0.49	0.31	1.06	0.19
	CP	30.11	50.20	6.68	3.97	2.36	1.91	0.48	1.09	0.58	0.32
	IVTD	59.73	20.88	2.94	7.73	2.67	1.44	0.48	1.05	0.46	0.29
	NDFD	75.94	2.90	7.23	2.55	4.24	3.00	0.50	0.75	0.55	0.19
	aNDF	33.29	45.87	8.35	3.29	2.71	1.56	0.31	1.25	0.46	0.41
Trinamix Static	ADF	74.94	10.20	2.82	2.63	1.33	2.96	1.53	0.70	0.27	0.59
	ADL	79.72	5.00	2.72	1.96	1.78	3.62	1.22	1.35	0.65	0.30
	CP	42.12	37.73	5.05	1.99	2.13	2.41	1.72	3.17	0.45	1.02
	IVTD	70.04	13.63	2.41	3.13	1.39	2.30	0.73	3.34	0.57	0.55
	NDFD	80.21	3.67	3.60	1.17	2.53	2.12	2.01	1.96	0.25	0.54
	aNDF	45.70	33.24	5.78	2.67	1.75	4.03	2.68	0.53	0.70	0.81

ADF—Acid Detergent Fiber, ADL—Acid Detergent Lignin, CP—Crude Protein, IVTD—in vitro Total Digestibility, NDFD—Neutral Detergent Fiber Digestibility, aNDF—actual Neutral Detergent Fiber.
